# Medication Safety Amid Technological Change: Usability Evaluation to Inform Inpatient Nurses’ Electronic Health Record System Transition

**DOI:** 10.1007/s11606-023-08278-1

**Published:** 2023-10-05

**Authors:** Carrie Reale, Deborah A. Ariosto, Matthew B. Weinger, Shilo Anders

**Affiliations:** 1 Center for Research and Innovation in Systems Safety, Department of Anesthesiology, Institute for Medicine and Public Health, and the Center for Health Services Research, Vanderbilt University Medical Center, Nashville, TN USA; 2 Department of Patient Care Services, Vanderbilt University Medical Center, Nashville, USA; 3 Department of Biomedical Informatics, Vanderbilt University Medical Center, Nashville, USA

**Keywords:** barcode medication administration, usability evaluation, EHR transition, patient safety

## Abstract

**Background:**

Electronic health record (EHR) system transitions are challenging for healthcare organizations. High-volume, safety–critical tasks like barcode medication administration (BCMA) should be evaluated, yet standards for ensuring safety during transition have not been established.

**Objective:**

Identify risks in common and problem-prone medication tasks to inform safe transition between BCMA systems and establish benchmarks for future system changes.

**Design:**

Staff nurses completed simulation-based usability testing in the legacy system (R1) and new system pre- (R2) and post-go-live (R3). Tasks included (1) Hold/Administer, (2) IV Fluids, (3) PRN Pain, (4) Insulin, (5) Downtime/PRN, and (6) Messaging. Audiovisual recordings of task performance were systematically analyzed for time, navigation, and errors. The System Usability Scale measured perceived usability and satisfaction. Post-simulation interviews captured nurses’ qualitative comments and perceptions of the systems.

**Participants:**

Fifteen staff nurses completed 2–3-h simulation sessions. Eleven completed both R1 and R2, and seven completed all three rounds. Clinical experience ranged from novice (< 1 year) to experienced (> 10 years). Practice settings included adult and pediatric patient populations in ICU, stepdown, and acute care departments.

**Main Measures:**

Task completion rates/times, safety and non-safety-related use errors (interaction difficulties), and user satisfaction.

**Key Results:**

Overall success rates remained relatively stable in all tasks except two: IV Fluids task success increased substantially (R1: 17%, R2: 54%, R3: 100%) and Downtime/PRN task success decreased (R1: 92%, R2: 64%, R3: 22%). Among the seven nurses who completed all rounds, overall safety-related errors decreased 53% from R1 to R3 and 50% from R2 to R3, and average task times for successfully completed tasks decreased 22% from R1 to R3 and 38% from R2 to R3.

**Conclusions:**

Usability testing is a reasonable approach to compare different BCMA tasks to anticipate transition problems and establish benchmarks with which to monitor and evaluate system changes going forward.

**Supplementary Information:**

The online version contains supplementary material available at 10.1007/s11606-023-08278-1.

## Introduction

An electronic health record (EHR) system transition is a massive undertaking for any healthcare organization. Technological change of this magnitude disrupts operations, consumes vast resources, and poses tremendous risk to patient safety.^1–6^ Even without major roadblocks, experienced clinicians may take years to return to pre-transition efficiency.^7^ If EHR features fail to support end-user needs, costly redesigns and repeated updates can extend the transition process.^7^ Unfortunately, stories of EHR implementation missteps abound.^8,9^ EHR transitions have increased as more organizations retire “homegrown” legacy systems or upgrade commercially available products.^5,10–15^ Switching EHR systems may pose unique risks and implementation concerns, but standards for ensuring safety during this process have not been established.^1,10,16^

EHR usability (i.e., efficiency, effectiveness, and satisfaction)^17^ has a profound effect on clinician burnout and patient safety.^1,18,19^ User-centered design (UCD), a human factors approach to system design that includes iterative end-user testing, is the gold standard for optimizing safety and usability.^20^ Despite certification requirements enacted to improve usability, there is considerable variability in the quality and extent of usability evaluation conducted by EHR vendors.^21^ A systematic review of EHR implementations found that projects often failed to incorporate human factors methods that would inform implementation decisions regarding user interface and training adjustments.^1^

Medication safety is a central concern during transitions as EHR systems are known contributors to medication errors.^19,22^ One organization reported a fivefold increase in medication safety reports in the 3 months following transition from a legacy EHR to a commercial system.^3^ Medication administration is a high-occurrence, time-consuming nursing task with frequent errors.^23^ While barcode medication administration (BCMA) technology has reduced errors,^24,25^ it has not eliminated them.^26,27^ An analysis of sentinel events found the EHR’s medication administration record (MAR) used for BCMA specifically contributed to errors.^22^ In addition, BCMA workarounds, such as bypassing the medication or patient armband scans, are well documented.^28–30^ These adaptions, whether problematic or pragmatic in nature, occur when the workflows envisioned by system developers clash with actual nursing practice.^31^

The goal of this study was to identify potential BCMA problems with nurses’ transition from a legacy EHR system to a commercially available EHR product configured for local use. We employed simulation-based comparative usability testing of BCMA tasks to assess progress during the transition. We collected quantitative performance and qualitative perception data to (1) establish baseline performance data for both BCMA systems, (2) pinpoint potential risks for safety critical tasks, (3) identify focus areas for superuser training and go-live support, and (4) offer evidence-based recommendations for enhanced configuration changes.

## Methods

Our simulation-based comparative evaluation included three rounds of data collection: (1) legacy system baseline performance (R1); (2) preliminary end-user performance in the new system prior to go-live (R2); and (3) follow-up evaluation 4 months post-implementation in the new system (R3). To ensure system stability across all three rounds and meaningful comparison of task performance, participants completed all tasks in a consistent training/testing version of the respective systems. Semi-structured interviews were conducted immediately after each session to capture nurses’ qualitative comments and identify themes related to system usability. This research was part of a larger study of EHR system usability approved by the organization’s Institutional Review Board in accordance with Human Research Protection Program guidelines.

### Participants

We recruited a convenience sample of 15 registered nurses at the study site to ensure participants represented varying levels of nursing experience and different inpatient care areas. Table 1 provides detailed participant demographics. Among the 15 individual participants, 12 completed the legacy system evaluation (R1), 14 completed the new system evaluation pre-implementation (R2), and 9 completed the new system evaluation post-implementation (R3). Eleven of the nurses completed both R1 and R2, and seven nurses completed all three rounds. All nine of the nurses in R3 completed at least one of the prior evaluation rounds. All participants reported using legacy BCMA and half (*n* = 7) had used another BCMA system. Among those who completed the R2 pre-implementation evaluation, most (*n* = 11) had no prior experience using the new EHR.

**Table 1:** Participant Demographics

Characteristic % (*n*)	All participants* n = 15	Round 1 *n* = 12	Round 2 *n* = 14	Round 3 *n* = 9
Gender				
Female	87% (13)	92% (11)	86% (12)	89% (8)
Male	13% (2)	8% (1)	14% (2)	11% (1)
Age				
20–29 years	40% (6)	50% (6)	36% (5)	33% (3)
30–39 years	33% (5)	25% (3)	36% (5)	44% (4)
40–49 years	27% (4)	25% (3)	29% (4)	22% (2)
Highest level of education				
Associate degree	7% (1)	8% (1)	7% (1)	0% (0)
Bachelor’s degree	73% (11)	75% (9)	71% (10)	67% (6)
Master’s degree	20% (3)	17% (2)	21% (3)	33% (3)
Years of nursing experience				
< 1 year	20% (3)	25% (3)	14% (2)	0% (0)
Between 1 and 2 years	13% (2)	17% (2)	14% (2)	33% (3)
Between 2 and 5 years	13% (2)	8% (1)	14% (2)	11% (1)
Between 5 and 10 years	13% (2)	17% (2)	14% (2)	11% (1)
> 10 years	40% (6)	33% (4)	43% (6)	44% (4)
Current patient care population				
Adult	60% (9)	50% (6)	57% (8)	44% (4)
Pediatric	33% (5)	42% (5)	36% (5)	44% (4)
Both	7% (1)	8% (1)	7% (1)	11% (1)
Current practice area				
Critical Care	33% (5)	33% (4)	36% (5)	22% (2)
Stepdown Unit	20% (3)	25% (3)	21% (3)	22% (2)
Acute Care/Med-Surg	47% (7)	42% (5)	43% (6)	56% (5)
Experience with baseline BCMA system				
< 1 year	20% (3)	25% (3)	14% (2)	33% (3)
Between 1 and 3 years	20% (3)	17% (2)	21% (3)	0% (0)
Between 3 and 5 years	13% (2)	17% (2)	7% (1)	22% (2)
> 5 years	47% (7)	42% (5)	57% (8)	44% (4)
Comfort with technology				
Among first of my peers to adopt	33% (5)	33% (4)	43% (6)	67% (6)
See how works for others first	60% (9)	67% (8)	43% (6)	22% (2)
Adopt only well-established tech	7% (1)	0% (0)	14% (2)	11% (1)
Reluctantly adopt new tech	0% (0)	0% (0)	0% (0)	0% (0)

^*^As reported at the individual’s first participation session

### Procedure

Individual performance-based usability testing sessions were conducted according to industry best-practices^20,32^ in the usability laboratory of the Center for Research and Innovation in Systems Safety (CRISS) on the VUMC campus approximately 4–6 months prior to go-live (R1 and R2) and 4 months after implementation (R3). Participants were scheduled for 2-h sessions for R1 and R3, and a 3-h session for R2. Because R2 was conducted prior to all staff completing formal house-wide training, in R2 only participants received 1 h of training at the start of their session. Standardized R2 training included four interactive modules from the EHR vendor that demonstrated how to (1) navigate the workspace; (2) administer medications; (3) document scheduled, overdue, missed, and held medications; and (4) document IV lines/fluids, and medication drips. Participants then completed self-guided hands-on practice with patient armband and medication barcodes. Since all participants were experienced legacy system users and had completed formal training in the new system by R3, no additional training was provided for these sessions.

A research nurse with usability testing experience facilitated all study sessions. Participants interacted with both systems on the same desktop computer in the laboratory with a comparable handheld barcode scanner. Test patients were created in the training environment (R1 and R2) or test environment (R3) of each system to match task scenarios. Patient armband and medication barcodes were provided on laminated cards standardized across all sessions. Morae® usability testing software digitally recorded the participant’s interactions, including all mouse movement and clicks, typing, and screens displayed during the tasks. A web-camera recorded each participant’s facial expressions and a microphone recorded audible system alerts (e.g., scanner beeps) and verbal comments.

We created realistic scenarios for common and problem-prone administration tasks based on the medication orders and barcodes used in nursing orientation legacy training (Appendix 1). We used the same standardized tasks with both systems. Differences in system capabilities required a few minor modifications to task details in the new system. Presentation order for the scenarios was varied to reduce potential order effects on performance. The tasks tested were as follows: (1) Hold/Administer (hold two and administer three medications while addressing alerts); (2) IV Fluids (switch existing fluids to a new order at a higher rate); (3) PRN Pain (administer medication and document pain assessment/score); (4) Insulin (administer complex insulin doses); (5) Downtime/PRN (document previously administered medications and administer PRN); and (6) Message (send a message to pharmacy to adjust insulin schedules). We incorporated more challenging tasks into the scenarios based on existing training foci for known administration difficulties as well as feedback from the implementation team. For example, the Hold/Administer task required the nurse to adjust the administered dose in response to a partial package dose alert, and the Insulin task required interpretation of a complex sliding scale based on a blood glucose value.

After all tasks, participants completed the System Usability Scale (SUS), a validated instrument to measure perceived usability and satisfaction.^33,34^ Scores range from 0-worst to 100-best. At the end of each session, we conducted brief semi-structured interviews to explore participants’ perceptions and experience using the BCMA systems beyond the specific tasks evaluated. Questions elicited positive and negative aspects of BCMA system use, usability issues encountered in practice, and perceptions of the transition process’ impact on workflow.

We analyzed key performance metrics for each session recording: task completion rates, safety-related errors (i.e., any error that has the potential to impact patient safety if it occurred in the real world), other use errors (i.e., an interaction difficulty or error not expected to impact patient safety), and task completion times. Task completion success criteria were based on critical task actions and the “six rights” of medication administration (right patient, medication, dose, route, time, and documentation). A task was categorized as a failure if the user finalized the task with the wrong information entered for one or more of these criteria, abandoned the task prior to completion, or required facilitator assistance to complete the task. Omissions of secondary task details (e.g., failed to scan a bar code) were counted as errors, not failures. Only times for tasks that were successfully completed were included in the task time analysis. To establish an additional benchmark and facilitate comparisons given minor system differences, we used keystroke-level modeling^35^ to estimate the time it would take an expert to complete each task (details provided in Appendix 2).

## Results

### Task Performance

**Figure 1:**
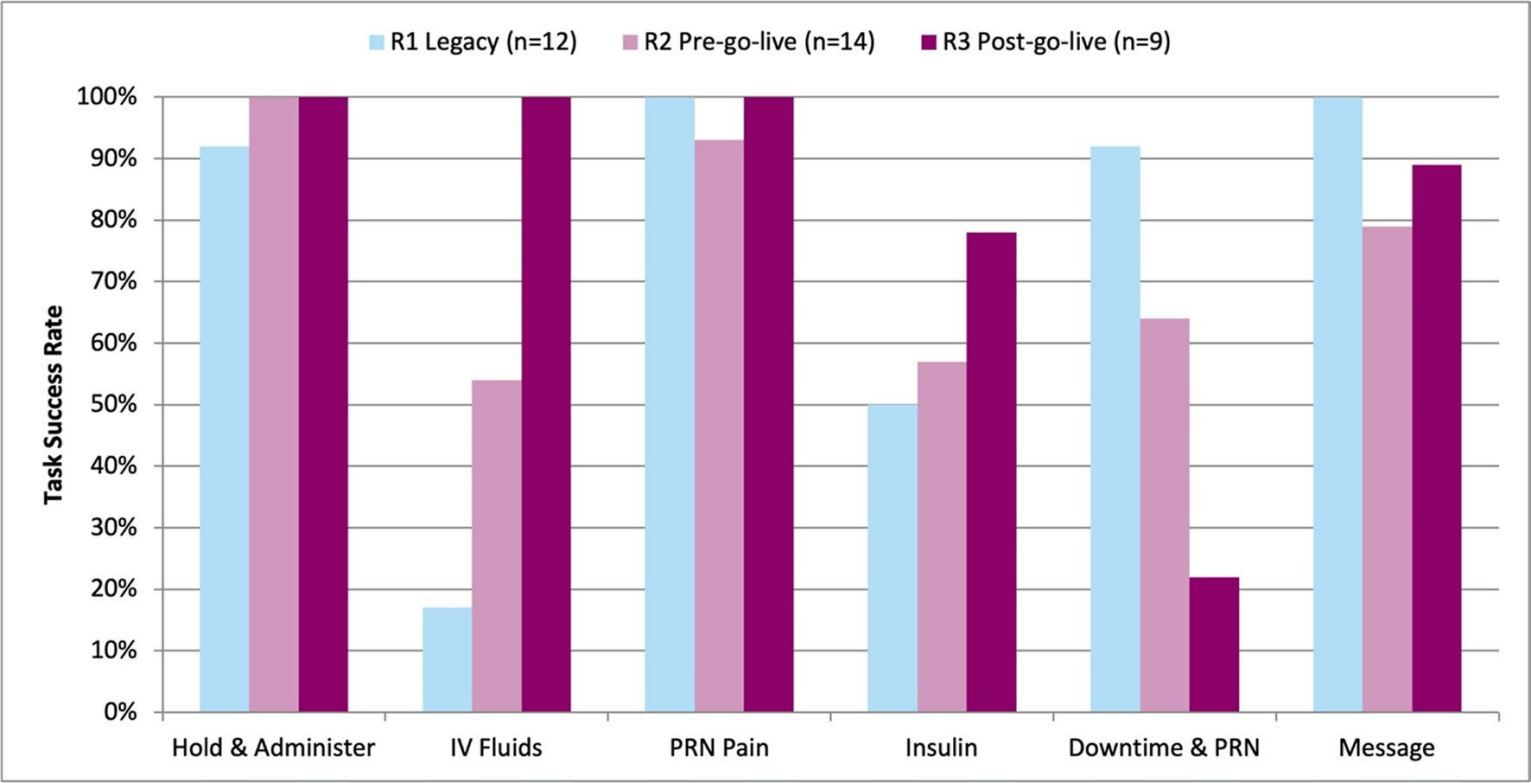
BCMA task success rates.

Table 2 provides detailed performance data by task and by round: task success and failure rates, safety-related errors, use errors, and task completion times. Figure 1 displays success rates by task and round. In most cases, nurses performed better on the post-implementation tasks compared to pre-implementation and legacy performance. Except for Downtime/PRN and Message tasks, success rates were maintained or improved post-implementation compared to baseline. Failure rates for the Downtime/PRN task markedly increased in R3, with successful completions dropping to a third of their R2 level. To confirm that participant dropout did not alter our conclusions, we performed a secondary analysis to compare the success rates for the seven nurses who completed all three rounds to the full sample and found performance was similar between groups (see Appendix 3).

**Table 2:** Task Performance

Task		Task successes % (*n*)	Task failures % (*n*)	Safety-related errors Mean ± SD (range)	Use errors Mean ± SD (range)	Task time* (min) Mean ± SD (range)
*Hold & Administer*	R1	92% (11)	8% (1)	0.3 ± 0.45 (0 − 1)	0.1 ± 0.29 (0 − 1)	2.16 ± 0.79 (1.14 − 3.74)
	R2	100% (14)	0% (0)	0.2 ± 0.43 (0 − 1)	2.1 ± 2.13 (0 − 8)	3.84 ± 1.23 (1.74 − 6.44)
	R3	100% (9)	0% (0)	0 ± 0 (0 − 0)	0.9 ± 1.17 (0 − 3)	2.12 ± 1.14 (1.17 − 4.67)
*IV Fluids*†	R1	17% (2)	83% (10)	0.8 ± 0.97 (0 − 3)	3.0 ± 1.6 (1 − 6)	3.23 ± 1.5 (2.17 − 4.29)
	R2	54% (7)	46% (6)	0.7 ± 0.95 (0 − 3)	1.8 ± 0.93 (0 − 3)	2.69 ± 1 (1.38 − 4.24)
	R3	100% (9)	0% (0)	0.2 ± 0.44 (0 − 1)	0.3 ± 0.5 (0 − 1)	1.23 ± 0.44 (0.74 − 1.92)
*PRN Pain*†	R1	100% (12)	0% (0)	0.1 ± 0.29 (0 − 1)	1.5 ± 1.17 (0 − 4)	2.13 ± 0.87 (1.24 − 4.20)
	R2	93% (13)	7% (1)	0 ± 0 (0 − 0)	0.4 ± 0.65 (0 − 2)	1.24 ± 0.71 (0.60 − 2.84)
	R3	100% (9)	0% (0)	0 ± 0 (0 − 0)	0.6 ± 1.13 (0 − 3)	1.01 ± 0.38 (0.53 − 1.65)
*Insulin*	R1	50% (6)	50% (6)	1.2 ± 0.83 (0 − 2)	1.8 ± 1.82 (0 − 5)	3.28 ± 1.06 (1.87 − 4.65)
	R2	57% (8)	43% (6)	0.6 ± 0.65 (0 − 2)	2.1 ± 1.82 (0 − 6)	3.50 ± 0.57 (2.63 − 4.24)
	R3	78% (7)	22% (2)	0.2 ± 0.44 (0 − 1)	1.2 ± 1.2 (0 − 4)	2.67 ± 0.76 (1.79 − 4.12)
*Downtime & PRN*	R1	92% (11)	8% (1)	0.2 ± 0.58 (0 − 2)	0.8 ± 1.11 (0 − 3)	1.97 ± 0.88 (0.82 − 3.92)
	R2	64% (9)	36% (5)	0 ± 0 (0 − 0)	1.4 ± 1.16 (0 − 4)	2.78 ± 0.84 (1.79 − 3.89)
	R3	22% (2)	78% (7)	0 ± 0 (0 − 0)	1.4 ± 1.13 (0 − 4)	1.22 ± 0.01 (1.21 − 1.23)
*Message*	R1	100% (12)	0% (0)	0.7 ± 0.49 (0 − 1)	0.5 ± 0.67 (0 − 2)	1.73 ± 0.94 (0.63 − 4.20)
	R2	79% (11)	21% (3)	0.9 ± 0.47 (0 − 2)	1.0 ± 1.04 (0 − 3)	2.25 ± 0.89 (1.07 − 4.05)
	R3	89% (8)	11% (1)	0.8 ± 0.83 (0 − 2)	0.2 ± 0.67 (0 − 2)	1.34 ± 0.48 (0.88 − 2.15)

^*^ Task times were only calculated for instances where the participant successfully completed the task

^†^ The IV Fluids and PRN Pain task details varied between EHRs due to differences in system capabilities, which limits the comparability of these task times

The IV Fluids and Insulin tasks saw the highest combined average error rates across rounds. Overall error rates generally decreased in R3, and specifically, the seven nurses who completed all three rounds demonstrated a 50% reduction in safety-related errors during the post-implementation tasks compared to pre-implementation, and 53% compared to legacy system performance. Table 3 summarizes the reasons participants failed to successfully complete tasks and the types of safety-related errors identified. Errors spanned all “six rights” for administration except route. Documentation (e.g., duplicate documentation of downtime administrations) and process-related errors (e.g., missed scans) occurred most frequently. Notably, a dosing error related to the display of sliding scale insulin (SSI) instructions occurred at least once in every round. Legacy system limitations prevented line breaks in the free text administration instructions field, forcing critical information like SSI guidance to be displayed in a single line of text. As a result, multiple nurses misinterpreted the series of colons, semicolons, and equal signs used to separate the blood glucose ranges and corresponding insulin units.

**Table 3:** Task Failure Reasons and Types of Safety-Related Errors

Task		Task failure reasons* (*n*)	Safety-related errors (*n*)
*Hold & Administer*	R1	• Did not administer meds due/completed hold meds portion of the task only (1)	• Manually changed multi-package dose vs. scanning both barcodes (2) • Did not administer meds (1)
	R2	N/A	• Manually changed multi-package dose vs. scanning both barcodes (3)
	R3	N/A	N/A
*IV Fluids*	R1	• Did not identify and/or document new rate (9) • Documented IV fluids as “Not Given” (1) • Did not administer IV fluids (1)	• Wrong intake volume documented (3) • IV fluids not scanned (2) • Did not identify new rate (2) • Patient not scanned (1) • Documented IV fluids as “Not Given” (1)
	R2	• Unable to create IV fluids stop action (5) • Unable to create IV fluids start action (1) • Wrong IV fluids order stopped (1) • Did not attempt to stop IV fluids (1)	• Did not indicate IV fluids had been stopped (6) • Wrong patient (1) • Wrong IV fluids stopped (1) • New IV fluids not started (1)
	R3	N/A	• Wrong intake volume documented (2)
*PRN Pain*	R1	N/A	• Patient not scanned (1)
	R2	• Did not document pain score (1)	N/A
	R3	N/A	N/A
*Insulin*	R1	• Wrong dose — did not administer second insulin order (3) • Wrong dose — misinterpreted sliding scale instructions (2) • Wrong site (2) • Did not cosign administration (1)	• Medication not scanned (6) • Did not administer second insulin order (3) • Wrong site documented (3) • Wrong dose — misinterpreted SSI instructions (2)
	R2	• Wrong dose — did not administer second insulin order (5) • Wrong site (1)	• Wrong dose — did not administer second insulin order (5) • Wrong dose — misinterpreted SSI instructions (1) • Wrong site documented (1) • Wrong patient (1)
	R3	• Wrong dose — misinterpreted sliding scale instructions (1) • Wrong site (1) • Unable to complete cosign process (1)	• Wrong dose — misinterpreted SSI instructions (1) • Wrong site documented (1)
*Downtime & PRN*	R1	• Documented as normal administration with no indication of downtime/paper MAR (1)	• Medication documented twice (1) • Deleted prior administration documentation (1)
	R2	• Documented as normal administration with no indication of downtime/paper MAR (5)	N/A
	R3	• Documented as normal administration with no indication of downtime/paper MAR (7)	N/A
*Message*	R1	N/A	• Did not send message for second insulin order (8)
	R2	• Wrong patient (1) • Wrong order (1) • Wrong schedule (1)	• Did not send message for second insulin order (10) • Wrong patient (1) • Wrong order — message sent on discontinued insulin order (1) • Wrong time requested in schedule change (1)
	R3	• Wrong schedule (1)	• Did not send message for second insulin order (5) • Wrong time requested in schedule change (1) • Due time changed on MAR for only 1 of 2 insulin orders (1)

^*^Participants may have more than one reason for failure per task

Inherent system differences limited comparability of task times in two tasks: PRN Pain (i.e., legacy system included full pain assessment documentation within the MAR; new system only included the pain score field) and IV Fluids (new system integrated infusion start/stop time documentation within the MAR and orders prefilled the flow rate; legacy system lacked both capabilities). For the remaining tasks, average completion times increased in R2 when nurses encountered the new system but decreased in R3 to levels at or below R1. Notably, participants who successfully completed tasks did so faster in every task post-implementation compared to legacy system performance. Among the seven nurses who completed all three rounds, average task times decreased by approximately 38% compared to pre-implementation performance and 22% compared to legacy performance.

### Satisfaction Scores

**Figure 2:**
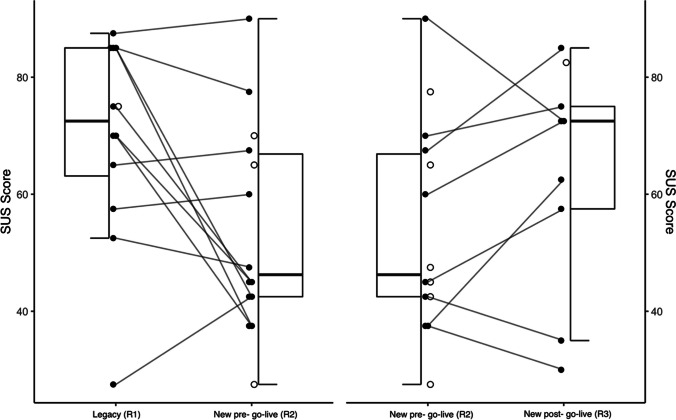
SUS scores across evaluation rounds. The figure displays the SUS score distribution for all available data at each time point, with the left panel comparing legacy system scores to the new system pre-go-live and the right panel comparing new system pre- and post-go-live. Closed points denote participants for whom data is available for paired data analyses, and the black connecting lines denote the individual change in SUS core. The boxes along the vertical lines denote the median (bold lines) and interquartile ranges (outer box). Using the Wilcoxon signed rank test to compare paired data gives a *p*-value of 0.068 for the left panel and 0.399 for the right panel.

Participant’s perceived ease-of-use and satisfaction with the systems were indicated by mean SUS scores of 69.6 (SD ± 17.4, range 27.5–87.5) in R1, 53.9 (SD ± 17.9, range 27.5–90) in R2, and 63.6 (SD ± 19.6, range 30–85) in R3. Figure 2 displays the SUS score distribution across rounds, highlighting the change in scores for nurses who completed both R1 and R2, or R2 and R3. While about half of the 11 nurses’ who completed both R1 and R2 rated both systems comparably, the decline in R2 SUS scores was largely due to a subset of nurses (*n* = 5) who rated the legacy system relatively high but the new system substantially lower pre-go-live.

### Interviews

Overall, most of the participants reported being excited about the new system, albeit nervous about the learning curve and how the change would impact workflows. Nurses consistently identified two factors that increased their confidence in a successful transition to the new system: (1) It is a proven system used nationally by many organizations, and (2) word-of-mouth from other nurses with prior new system experience has been positive.

Thematic analysis of the qualitative data uncovered three major usability themes expressed by nurses: (1) increased documentation burden; (2) excessive alerts/prompts; and (3) inefficient flowsheet design. Participants also identified several areas of dissatisfaction with BCMA processes in the new system that fell outside the scope of this evaluation (e.g., heparin co-sign processes, medication dispensing machine display issues, problematic blood administration documentation workflows). A detailed list of participants’ comments is provided in Appendix 4.

### Informing Stakeholders

Stakeholders (nurse executives, informatics leaders) received a detailed review of the findings along with training emphases and targeted interface enhancement suggestions that would potentially address some of these priority areas following R2 and R3. Table 4 shows examples of the type of actionable recommendations informed by our quantitative and qualitative findings provided to transition leadership. As modifications to a user interface within a complex system can result in unintended consequences, additional post-implementation user testing was recommended to validate the effectiveness of the recommendations in a real-world use setting.

**Table 4:** Example Interface Design, Workflow, and Training Recommendations

Finding	Recommendation	Training emphasis
***Interface design recommendations***		
**MAR:** Each medication row in the MAR uses a substantial amount of screen space, resulting in frequent vertical scrolling. When a patient has more than a few meds ordered, users cannot see all meds due on a single screen. This increases risk for missed meds that fall above or below the visible area.	Move the “Due/Overdue” tab to the first tab position, making it the default screen upon entering the MAR instead of the “All” meds tab. This will decrease the number of meds displayed to only those requiring action and better align with nurses’ most frequent MAR task: administering due meds.	Emphasize the “Refresh” icon at the top of the MAR section when working in the Due/Overdue tab. This action removes completed meds from the screen and consolidates any remaining due meds at the top of the screen. Demonstrate that a consolidated list of due meds can also be displayed by hovering the cursor over the time at the top of a MAR column in the main MAR screen. However, this feature is not available while administering meds.
**MAR:** When a medication is scanned, the administration window hides the main MAR screen and additional meds due now are no longer visible. Users cannot move or navigate away from this screen without discarding or saving their work in progress. Note: This is a change from the current workflow.	Consider adding a consolidated version of the “Due Meds” report (currently available in Patient Lists lower frame) to the patient chart side bar index (right of screen).	Make nurses aware of the “MAR Report” (in menu bar at top left of MAR section). This report is accessible while in the administration screen and shows all med orders with associated due times.
***Workflow/process recommendations***		
**Viewing meds due for multiple patients:** There is no apparent at-a-glance visual display of meds due for multiple patients over time. Nurses indicate heavy reliance on the “To Do” screen in their current workflow. This tool is used for planning at the start of shift as well as adjusting workflow and tracking med activity throughout the shift.	Confirm that there is not an existing tool available that meets this important user need. If not, consider prioritizing development of a comparable report during optimization (i.e., provides a condensed view that allows for visualization of meds due for their entire patient assignment across the shift).	
**Meds requiring co-sign:** Many nurses viewed dual sign off in the moment as a reasonable workflow change in most cases. It was seen as an important safety enhancement and will relieve them from the burden of double checking that the other person has done it or remembering to do it themselves after the fact. However, some described circumstances (e.g., low unit staffing levels; simultaneous code/bedside procedures taking priority) where they are not able to physically get a second nurse into the room in current state. This hard stop may force nurses to decide between delaying patient care or performing BCMA processes after administration (workarounds).	Carefully review the dual sign off medication list to ensure it only includes those medications where the risk outweighs the added workload. Investigate whether an emergency override is possible for dual sign off, or if there is an alternative process that allows nurses some flexibility in extreme cases. Ideally, an alternative or override option would be available for these circumstances that captures the override reason for close monitoring of potential barriers.	
***Additional training recommendations***		
**MAR:** It is not obvious that the MAR allows multiple meds to be scanned and administered as a batch. Nurses assumed each med had to be scanned and accepted individually, which contributed to increased task times and more frequent switching between input methods (scanner, keyboard, mouse).		Demonstrate the option of scanning several meds into the administration screen and accepting them as a batch. Note: This workflow was not covered in the vendor’s standard MAR-related learning modules.
**MAR:** Users struggled to create a stop action for continuous infusions in the MAR.		Reinforce this interaction method (This was shown in the vendor’s learning module; however, many users could not remember how to do it). Demonstrate that stop/restart actions can also be created from the Flowsheet activity in the IVF’s intake row.

The recommendations attempted to account for available local configuration options and limitations in our ability to modify the vendor’s system. These recommendations were not intended to disrupt the transition process, but rather to minimize short-term risk through improved awareness and training, and to suggest interface improvement opportunities during optimization. Similarly, we highlighted areas where nurses struggled or expressed confusion during the evaluation, as these represent areas where additional reinforcement during training and early implementation may be helpful. These needed not alter existing training curricula but could be effectively disseminated in supplemental forms (e.g., training tips distributed to superusers or additional practice scenarios for self-study in the system “playground”).

## Discussion

Findings suggest that in less than 6 months post-go-live, these nurses had adapted to the new BCMA system and experienced enhancements in efficiency and effectiveness for the specific tasks evaluated. This study confirmed known legacy system problems (IV fluids, SSI), identified new system problems (downtime, messaging), and provided quantified performance data (error rates, time) against which to benchmark future system and workflow changes. Task time data helped inform expectations for learning curve workload increases during the initial go-live phase.

The study team made formal recommendations for system, workflow, and training changes; however, measuring the extent to which they were adopted and their impact on real world performance was outside the scope of this study. Go-live is just one point in time for a dynamic EHR ecosystem that is continuously evolving. This usability study not only provided transition insights into the new system, but an awareness of where the legacy system was not performing as expected. While it is important to prepare for transitions, it is prudent to use insights gained and study benchmarks to monitor and tune systems affected by modifications, updates, and user experiences going forward. Post-implementation medication error reporting studies^3^ are needed to help connect the dots between usability problems and medication errors.

### Limitations

This study has limitations. It was conducted at a single academic medical center with extensive experience in clinical informatics and usability evaluations.^36–39^ We were only able to test a subset of BCMA tasks performed by nurses. Future research could expand the number of user groups, study sites, and range of tasks evaluated.

### Implications/Future Directions

Application of usability testing methods to the EHR transition process can yield important evidence-based insights to inform end-user safety during this period of immense change. EHR vendors should incorporate usability evaluation of high-risk tasks into their customer implementation roadmaps. Adding formal usability requirements to EHR Requests for Proposals (RFP) could improve vendors’ current suboptimal approach to usability^21^ and shift industry expectations from a focus on user acceptance testing and whether the system is “working as designed by vendor” to high-quality user-centered design and “exceeding client requirements.” This study also identified serious usability issues in the legacy EHR while collecting baseline data. Thus, even after substantial experience using a system, design flaws in a well-established EHR can continue to create significant potential for errors and inefficiencies. Incorporating multiple human factors methods, including usability testing, heuristic analysis, and risk analysis, into implementation protocols for regular system updates may be necessary to ensuring safe use beyond the transition period. Healthcare organizations should freely share their evaluation findings, including all safety and usability issues uncovered, whether addressed through local configuration changes or requiring vendor intervention, to help improve the collective usability of and establish evaluation standards for commercial EHR products for all users.

### Supplementary Information

Below is the link to the electronic supplementary material.

Supplementary file1 (DOCX 300 KB)

## Data Availability

The data that support the findings of this study are available from the corresponding author, CR, upon reasonable request.
