# Supplementary material for: Medication Safety Amid Technological Change: Usability Evaluation to Inform Inpatient Nurses’ Electronic Health Record System Transition
Source: J Gen Intern Med. 2023 Oct 5;38(Suppl 4):982–90. doi: 10.1007/s11606-023-08278-1 (PMC10593701; doi:10.1007/s11606-023-08278-1)
Supplement: Supplementary file 1 — Supplementary file1 (DOCX 300 KB) [file 11606_2023_8278_MOESM1_ESM.docx]

Appendices

Appendix 1. Task Scenarios

*Legacy System:*

Scenario: Hold & Administer (Holly – Part 1)

You are caring for Holly, a female patient admitted for surgical repair of a closed femoral shaft fracture. She has a history of type 2 diabetes, hypertension, and Tourette’s. You check her glucose and it’s 57. You notify the physician, who instructs you to hold the entire 10:00 AM insulin dose, get her some juice and a snack instead of giving D50, and then recheck her glucose in 30 minutes. You get her something to eat and administer the rest of the meds due now.

Document administration of her 10:00 AM medications accordingly.

Scenario: IV Fluids (Holly – Part 2)

Imagine it’s now 30 minutes later…you recheck Holly’s glucose and it’s 74. She had Lactated Ringer’s infusing at 100 ml/hr through a peripheral IV in her left arm. The physician has discontinued the LR and placed a new order for D5 ½ NS with potassium at a higher infusion rate.

Document administration and the rate change for the new IV fluids.

Scenario: PRN Pain (Holly – Part 3)

Holly’s post-op PCA pump was stopped earlier this morning. You respond to her call bell and discover she is having throbbing pain in her left leg around the surgical site. She reports her current pain level is a 6 out of 10, but she wants this to be a 3 or below. You apply an ice pack and prepare to give a prn.

Document administration of her prn pain medication.

Scenario: Insulin (Morgan – Part 1)

You are caring for Morgan, a female patient admitted for surgical repair of a ruptured spleen following a motor vehicle accident. She has been hypertensive, hyperglycemic and agitated. You check her glucose, and it is 262. You administer her insulin per orders, subq in the right lower abdomen.

Document the double check by a second RN and administration of her 10:00 AM insulin doses accordingly.

Scenario: Downtime & PRN (Morgan – Part 2)

Imagine it’s now a little later in your shift. The computer has been down. You administered the rest of Morgan’s scheduled 10:00 AM meds, documenting this on the paper MAR per downtime procedures. Orange alert has been cancelled and the system is back up. The patient complains of severe nausea, so you administer a prn dose of ondansetron (Zofran).

Document administration of this prn medication and update the remaining 10:00 AM scheduled meds to indicate that they were previously administered during downtime.

Scenario: Message (Lori)

You are caring for Lori, a female patient with type 2 diabetes. The patient needs to have her scheduled insulin doses delivered before meals, which means the due times should be 7:00 AM, 12:00 PM, and 6:00 PM daily.

Send a message to the pharmacy to have the insulin schedule adjusted accordingly.

*New System:*

Scenario: IV Fluids (Holly – Part 1)

You are caring for Holly, a female patient admitted for surgical repair of a closed femoral shaft fracture. She has a history of type 2 diabetes, hypertension, and Tourette’s. She had ½ normal saline with potassium chloride infusing at 75 ml/hr through a peripheral IV in her left arm. The physician has asked you to switch her fluids to D5 ½ NS + KCL at a higher infusion rate, as ordered.

Document administration of a new bag of the D5 ½ NS + KCL, and that you stopped the ½ NS + KCL infusion.

Scenario: Hold & Administer (Holly – Part 2)

You check Holly’s glucose and it’s 57. You notify the physician, who instructs you to hold the entire 10:00 AM insulin dose, get her some juice and a snack instead of giving D50, and then recheck her glucose in 30 minutes. You get her something to eat and administer the rest of the meds due now.

Document administration of her 10:00 AM medications accordingly.

Scenario: PRN Pain (Holly – Part 3)

Holly’s post-op PCA pump was stopped last night. You respond to her call bell and discover she is having throbbing pain in her left leg around the surgical site. She reports her current pain level is a 6 out of 10, but she wants this to be a 3 or below. You apply an ice pack and prepare to give a prn.

Document administration of her prn pain medication.

Scenario: Insulin (Morgan – Part 1)

You are caring for Morgan, a female patient admitted for surgical repair of a ruptured spleen following a motor vehicle accident. She has been hypertensive, hyperglycemic and agitated. You check her glucose, and it is 262. You administer her insulin per orders, subq in the right lower abdomen.

Document the double check by a second RN and administration of her 10:00 AM insulin doses accordingly.

Scenario: Downtime & PRN (Morgan – Part 2)

Imagine it’s now a little later in your shift. The computer has been down. You administered the rest of Morgan’s scheduled 10:00 AM meds, documenting this on the paper MAR per downtime procedures. Orange alert has been cancelled and the system is back up. The patient complains of severe nausea, so you administer a prn dose of ondansetron (Zofran).

Document administration of this prn medication and update the remaining 10:00 AM scheduled meds to indicate that they were previously administered during downtime.

Scenario: Message (Lori)

You are caring for Lori, a female patient with type 2 diabetes. The patient needs to have her scheduled insulin doses delivered before meals, which means the due times should be 7:00 AM, 12:00 PM, and 6:00 PM daily.

Send a message to the pharmacy to have the insulin schedule adjusted accordingly.

Appendix 2. Keystroke Level Modeling

To establish an additional time benchmark and facilitate task time comparisons, we used keystroke-level modeling^35^ to estimate the time it would take an expert to complete each task. Inherent system differences limited comparability of task times in two tasks: PRN Pain (i.e., legacy system included full pain assessment documentation within the MAR; new system only included the pain score field) and IV Fluids (new system integrated infusion start/stop time documentation within the MAR and orders prefilled the flow rate; legacy system lacked both capabilities).

An “expected task pathway” outlining every mouse click and keyboard stroke required to complete the task was determined based on system training protocols and validated by a nurse informaticist familiar with the workflows in both systems. Using Morae^®^ to capture performance, another nurse informaticist experienced with the legacy system, trained on the new system, and familiar with the expected task pathways performed each task three times without making errors. Task times were averaged to obtain an expected completion time in each system.

The estimated task times provided an alternative benchmark for comparing actual participant performance across tasks and to reduce potential variances associated with errors and learning curves (see Figure 3). Nurses spent more time than expected completing all six tasks in the legacy EHR despite experience using that system. As anticipated, R2 actual times exceeded expected times, however, R3 actual times were considerably closer to expected times than R1 times.

Figure 3. Actual task times compared to expected task times by EHR system


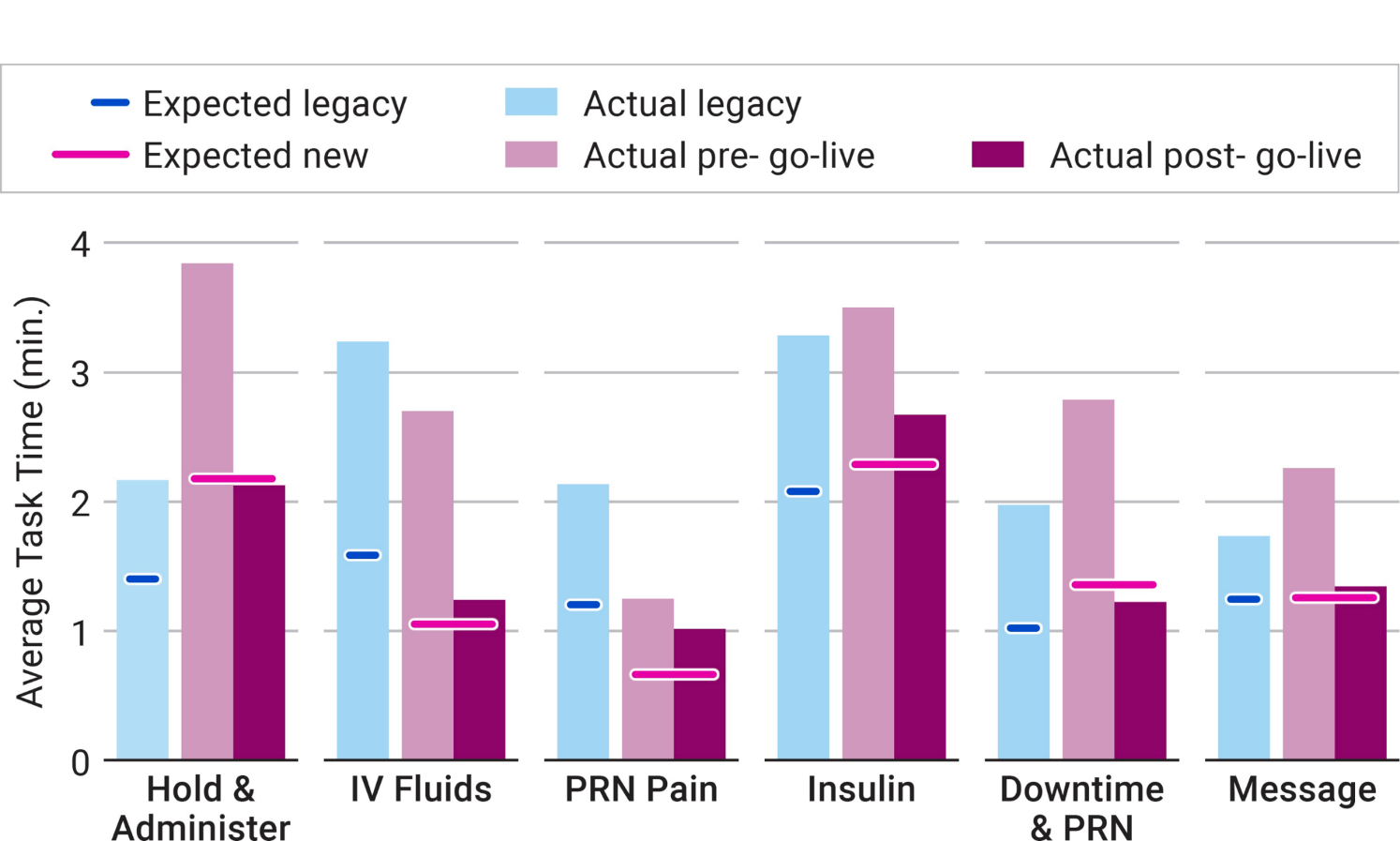


Appendix 3. Analysis of the impact of participant dropout on task success rates

To confirm that participant dropout did not alter our conclusions, we performed a secondary analysis to compare the success rates for the seven nurses who completed all three rounds to the full sample of nurses. As shown in Figure 4 below, task performance results among both groups were similar and did not change our conclusions.

Figure 4. Success rate by task and round

R1=Legacy system

R2=New system pre-go-live

R3=New system post-go-live

Appendix 4. Qualitative feedback

Table 5. Nurses’ qualitative comments about the new BCMA system

| **Satisfying features and characteristics** | **Dissatisfying features and characteristics** |
| --- | --- |
| - Improved integration between nursing specific tools and other EHR components (9) - Improved pharmacy message functionality (e.g., easily accessible, displays message history, prioritization flags) (6) - Automated intake calculation capabilities (6) - Improved continuous IV fluid functionality (e.g., displayed on MAR, stop/restart capabilities) (5) - Colored hourly due/overdue boxes easy to see (5) - Scanning patient first is safer for patient (4) - Easy to see last admin time/next due time (2) - Won’t have to follow up to make sure cosign gets documented (2) - MAR tabs group meds by category (2) - Relevant vital signs integrated with administration screen (2) - Easy to move in and out of patient charts (1) - Large activity buttons for most frequent activities (1) - Scanned meds clearly distinguished from meds not scanned (1) - Discontinued/completed meds displayed on MAR (1) - Cosign with the same ID/password as login (1) - Easy to document that a med was held (1) - Quick pick buttons to document pain score (1) - Both brand name and generic displayed (1) - Med education can be documented in MAR (1) - Override pulls can be linked to MD order in MAR (1) | - Too much information on the screens (7) - Seems harder to plan for shift/no way to quickly see med schedules for my entire assignment (5) - Must scroll up and down to see all meds on MAR (5) - Other meds are not visible while scanning (4) - Separate base/sliding scale insulin orders is confusing/error prone (3) - Unable to see entire 12-hr shift on MAR screen (3) - Scanning patient first will be difficult in the real world (e.g., isolation/precaution rooms with no computer, sleeping patients at night) (2) - Requires too many clicks (2) - Too many colors/nondescript icons (2) - Pain charting in MAR doesn’t allow for full assessment (2) - IV assessment fields not on MAR screen (1) - Can’t scroll horizontally 1 hour at a time on MAR (1) - Start/stop IVF functionality increases workload (1) - Cosign hard stop will be difficult in the real world (e.g., staffing issues) (1) - Due Meds report is difficult to interpret (1) |
